# Four- and five-photon upconversion lasing from rare earth elements under continuous-wave pump and room temperature

**DOI:** 10.1515/nanoph-2022-0360

**Published:** 2022-08-15

**Authors:** Bo Jiang, Yuchan Hu, Linhao Ren, Han Zhou, Lei Shi, Xinliang Zhang

**Affiliations:** Wuhan National Laboratory for Optoelectronics, Huazhong University of Science and Technology, Wuhan 430074, China; Optics Valley Laboratory, Wuhan 430074, China

**Keywords:** multiphoton upconversion, optical microcavity, rare earth, upconversion laser, whispering gallery mode

## Abstract

Benefited from abundant long-lived intermediate energy levels of rear earth elements, large anti-Stokes lasing can be realized by multi-photon upconversion processes, which does not demand rigorous phase match and ultrahigh pump power. Here, we have fabricated an Er-doped silica microsphere with an ultrahigh intrinsic quality factor of 1.2 × 10^8^. By continuous-wave (CW) excitation at 1535 nm, four- and five-photon upconversion lasers are achieved simultaneously under room temperature, in which the lasing thresholds are estimated as 176 and 600 μW, respectively. Beside the ultralow thresholds, the microlaser also exhibits good stability of lasing intensity for practical applications. The four- and five-photon upconversion lasing from rare earth elements have not been separately demonstrated under CW pump and room temperature until this work. This demonstration provides a prospect to realizing high-performance short-wavelength laser by pumping low-energy photons.

## 1 Introduction

Upconversion luminescence is an effective mechanism for achieving large anti-Stokes lasing radiation by pumping low-energy photons [1]. It can be realized by harmonic generations [2–5], parametric oscillation [6, 7], and simultaneous multi-photon absorption [8, 9]. Nevertheless, harmonic generation and parametric oscillation require rigorous phase match, and simultaneous two-photon absorption requires ultrahigh energy density. In contrast, rare earth (RE) based upconversion luminescence can be easily realized through their long-lived intermediate energy levels [10, 11].

Higher-order upconversion lasers usually demand higher pump power to inverse population and overcome extreme scattering loss at short wavelength. Therefore, pulsed laser pump [12, 13] and cryogenic environment [14, 15] were employed to avoid optical and thermal damages. By employing 980 nm pulsed laser pump, five-photon upconversion lasers have been achieved in NaYbF_4_:Tm@NaYF_4_ doped microbottles [16], NaYbF_4_:Gd/Tm@NaGdF_4_@CaF_2_:Ce nanoparticle [17] and LiYbF_4_:Tm @LiYbF_4_@LiLuF_4_ nanocrystal based microdisks [18]. However, these high-order upconversion lasers are demonstrated under pulsed pump, which extremely limits their practical applications. By employing continuous-wave (CW) pump, three-photon upconversion lasers have been achieved by Tm-doped nanoparticle microsphere [19] and silica-coated β-NaYF_4_:Yb^3+^, Tm^3+^ microsphere [20], respectively. Besides, three-photon upconversion CW lasers are also realized by Tm- [21] and Er- [22, 23] doped silica microtoroids with the sol-gel technology. However, a higher-order upconversion based CW laser under room-temperature has not been reported, to the best of our knowledge. Among the RE elements, erbium (Er) provides homogeneous energy differences of about 6400 cm^−1^, which matches well with the absorption of 1540 nm photons. Eleven-order spontaneous upconversion emission of Er has been reported, indicating its potential for high-order upconversion lasers [24, 25].

In this work, we fabricated an Er-doped silica microsphere with an ultrahigh intrinsic quality (Q) factor of about 1.2 × 10^8^, achieving four- and five-photon upconversion lasers under 1535 nm excitation, where the thresholds are as low as 176 and 600 μW, respectively. The five-photon upconversion laser should be attributed to the highest order rare-earth based upconversion laser under room temperature and CW pump. This microlaser exhibits relatively good stability over 120 min duration, indicating its potential application in short-wavelength laser sources.

## 2 Results and discussion

The Er-doped microsphere fabrication employs polymethyl methacrylate (PMMA) for avoiding obvious ion clusters. Firstly, the Er-PMMA solution is made by mixing erbium nitrate pentahydrate (Er (NO_3_)_3_·5H_2_O, 99.9%, Macklin), polymethyl methacrylate (PMMA, TCI), and acetone (AR, Shanghai Hushi) with a weight ratio of 1:5.177:136.3. Secondly, we process a standard single-mode optical fiber to form a microbottle cavity by carbon oxide (CO_2_) fabrication platform (the microbottle fabrication detail refers to reference [26]). Thirdly, dip the microbottle into the prepared solution and then draw it out. After evaporation, an Er-PMMA film will homogeneously form on the surface of the microbottle. Lastly, reflow the microbottle to form a microsphere by CO_2_ fabrication platform. During the reflow process, the PMMA will be fully eliminated and the rare earth element will be doped into the interior of the microsphere.

**Figure 1:**
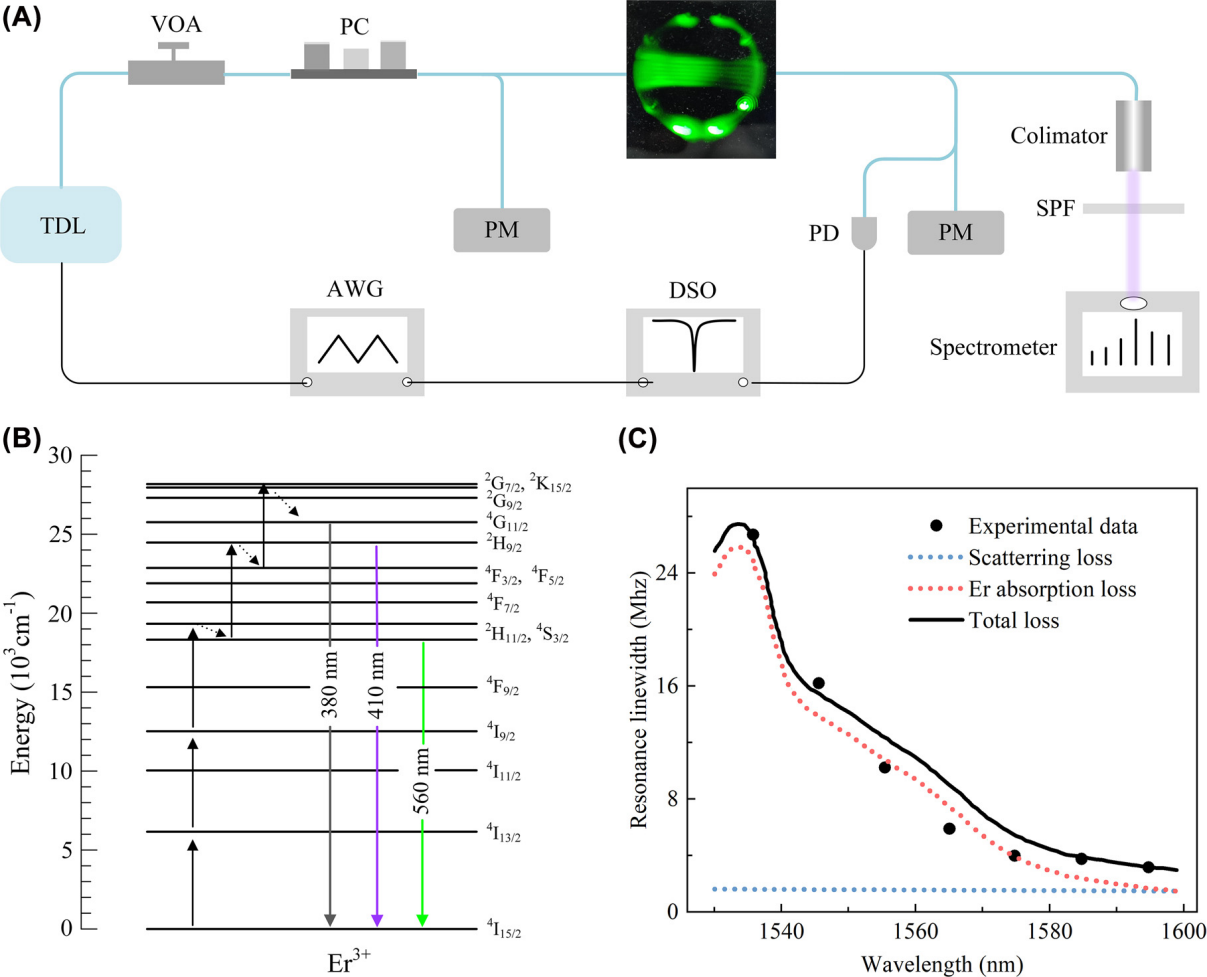
Four- and five-photon upconversion CW lasing at room temperature. (A) Experimental set-up. AWG, arbitrary waveform generator; TDL, tunable diode laser; VOA, variable optical attenuator; PC, polarization controller; PM, power meter; SPF, short-pass filter; PD, photodetector; DSO, digital storage oscilloscope. (B) Energy level diagram and proposed emission mechanisms of four- and five-photon upconversion processes. The dotted arrows represent the NR process. The upward full arrows represent the upward transition induced by the photon excitation and ET processes. (C) Resonance linewidth evolution in a wavelength range covering six FSRs. The black fitting curve represents the total resonance linewidth induced by the absorption loss of the gain materials and the surface scattering loss.

As shown in Figure 1A, an Er-doped silica microsphere cavity with a diameter of about 56 μm is coupled with an optical microfiber with a diameter of about 2 μm. The microsphere is pumped by a tunable diode laser (Toptica, CTL 1550) operating around 1535 nm, which is corresponding to the absorption cross-section peak of Er. By finely tuning the pump wavelength continuously, the pump wavelength is stabilized in the blue detuning regime of a resonance mode. And the power injected into the cavity is defined as the pump power. Upconversion emission is coupled to a spectrometer (Horiba, iHR-550) by a collimator, and detected by an electric cooled silicon array charge-coupled device (Horiba, 1024X256-OE), employing a grating of 1800 groove/mm blazed at 450 nm. The CCD image of the pumped microsphere with green rings shows the distribution of the whispering gallery modes (WGMs). The green luminescence is a major upconversion emission in Er and has high response for CCD detection; therefore, green light is dominated in the CCD image. Resonance linewidth measurement is performed using the tunable diode laser sweeping over the resonance modes with a speed of 4.8 GHz/ms. And the signal power is below 10 µW to avoid the resonance linewidth narrowing induced by the thermal effect and saturated absorption of Er.

The upconversion processes in Er are initiated by the ground state absorption (GSA) and further promoted during the excited state absorption (ESA), energy transfer (ET) and nonradiative relaxation (NR) processes [13]. As shown in Figure 1B, under 1535 nm photon excitation, the G11/24 and H9/22 states are populated, which can be described by the following upconversion process: I15/24→GSAI13/24→ESA/ETI9/24→ESA/ETH11/22→NRS3/22→ESA/ETH9/22→NRF3/24→ESA/ETG7/22→NRG11/24 . The G11/24 and H9/22 states transit to the ground state will emit the 380 (ultraviolet)- and 410 (violet)-nm photons, which corresponds to five- and four-photon upconversion processes [23, 24], respectively. Here, we measured the total optical loss of a certain resonance mode over six free spectrum ranges (FSRs) with a spacing of one FSR. The coupling-loss-excluded linewidth of the transmission spectrum represents the total decay rate of the resonance mode. The total optical loss of the microcavity can be mainly divided as the Er absorption loss [27], the surface scattering loss [28], and the water absorption loss [29]. In this characterization, the water absorption loss is not separated from the total loss (The detailed analysis is shown in Section I of the Supplementary Material). The fitting result shows that the Er absorption loss and surface scattering loss induced linewidths at 1533 nm are 25.85 and 1.62 MHz, respectively, as shown in Figure 1C. Therefore, it can be inferred that the intrinsic Q factor of the cavity is estimated as 1.2 × 10^8^, and the effective doping concentration of Er is about 3 × 10^17^/cm^3^. This effective doping concentration takes the overlap between the actual distribution of Er and the resonance mode into consideration [30]. The doping process is realized by the thermal diffusion of Er from the surface to the interior of the microsphere, which depends on the temperature and the diffusion time. The expected doping concentration around the cavity surface should be far larger than the effective doping concentration. It is well known that the ET process is quadratically enhanced by the doping concentration; a higher doping concentration will achieve a higher upconversion efficiency [31].

**Figure 2:**
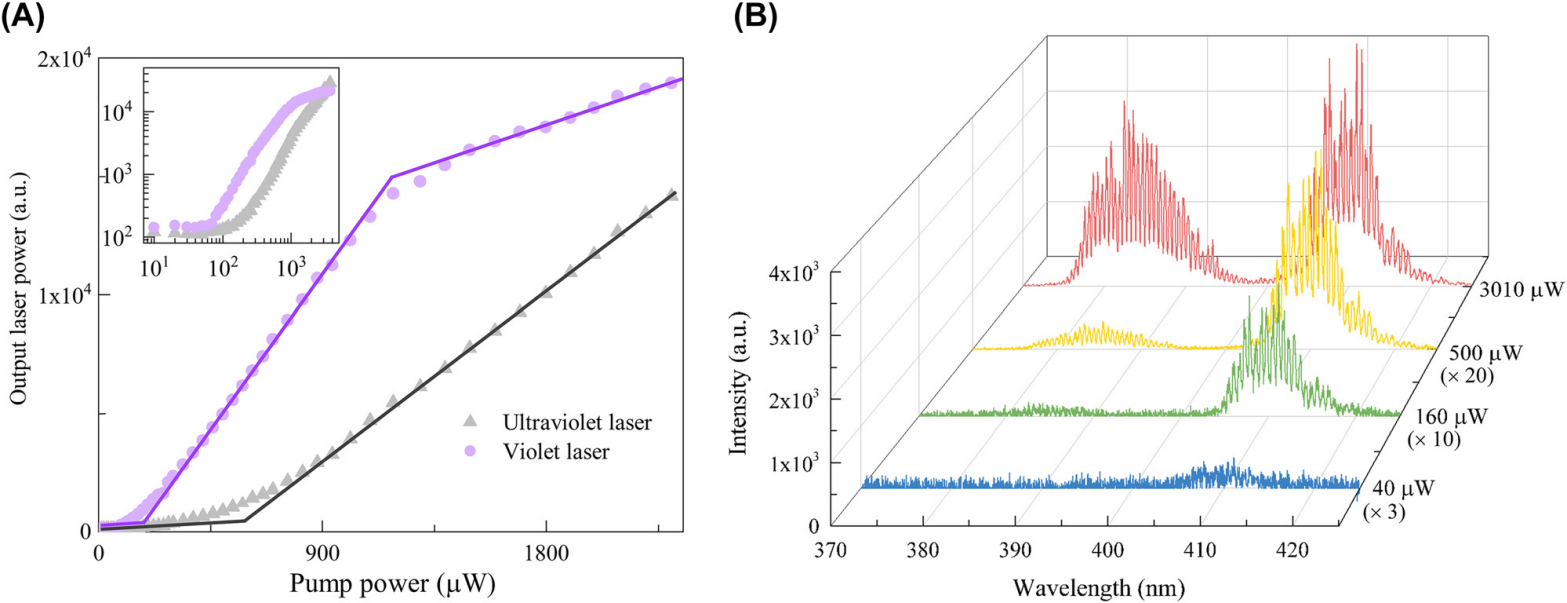
Lasing characterizations. (A) Emission intensities of ultraviolet and violet lasers versus the pump power in linear scale. The inset shows the plot in log–log scale. Their emission intensities are integrated from 375 to 395 nm and 400 to 425 nm, respectively. (B) Lasing spectrum evolution with the increased pump power.

Our experiment employs the 1535-nm-wavelength laser to pump the microsphere cavity resonantly. Benefited from the ultrahigh intrinsic Q factor of the microsphere, the pump power is extremely enhanced, achieving high pump efficiency for a low lasing threshold [32–35]. Ignoring the absorption loss of the gain materials, at 1 mW injected power, the resonant power in the cavity is enhanced up to about 100 W, which corresponds to an ultrahigh effective energy density of about 74 mW/μm^3^. As this microsphere lases with multiple modes, and the lasing modes are not well distinguished by the spectrometer. The output intensity is integrated over the whole emission band instead of a certain lasing mode. Figure 2A shows the linear plots of the output intensity versus the pump power. By fitting the curves by straight lines, the lasing thresholds of the ultraviolet and violet lasers in linear scale are estimated to be 600 and 176 μW, respectively. The estimated lasing thresholds in log–log scale are 670 and 187 μW, respectively [36] (the detail is shown in Section II of the Supplementary Material). The ultraviolet laser has a higher lasing threshold than that of the violet laser, as the ultraviolet laser requires one more absorbed photon for upconversion emission. The thresholds of the four- and five-photon upconversion lasing are even lower than those in previous works on three-photon upconversion lasers [22]. It is worth noting that RE based four- and five-photon upconversion lasers are first realized under room temperature and CW pump. Shown in Figure 2B is the overall emission spectrum of the microsphere with the increased pump power. At an excitation power of 3010 μW, the multi-mode lasing is clearly exhibited. And the spacings between the lasing modes are 0.55 and 0.63 nm for the ultraviolet and violet lasers, respectively, which is well matched with the theoretical FSRs. We have further measured the optical spectra in the pump wavelength band. Except for upconversion lasers, Er-based downshifting laser, Raman laser, and four-wave mixing are also observed. The lasing threshold for Er-based downshifting laser is about 0.4 μW. Raman laser is suppressed by Er induced absorption, achieving a relatively high lasing threshold of 430 μW (the details are shown in Section III of the Supplementary Material). Short-wavelength multi-mode lasing usually occurs in ultrahigh-Q WGM microcavites [21, 22, 37], [38], [39]. Firstly, the microcavity with a small FSR at short wavelengths supports many resonance modes over the span of the emission wavelength. Secondly, to realize lasing, the gain material absorption loss and the intrinsic cavity loss must be overcome, achieving a positive small-signal gain. However, in an ultrahigh-Q microcavity, its intrinsic cavity loss is easy to be overcome over a large wavelength span.

**Figure 3:**
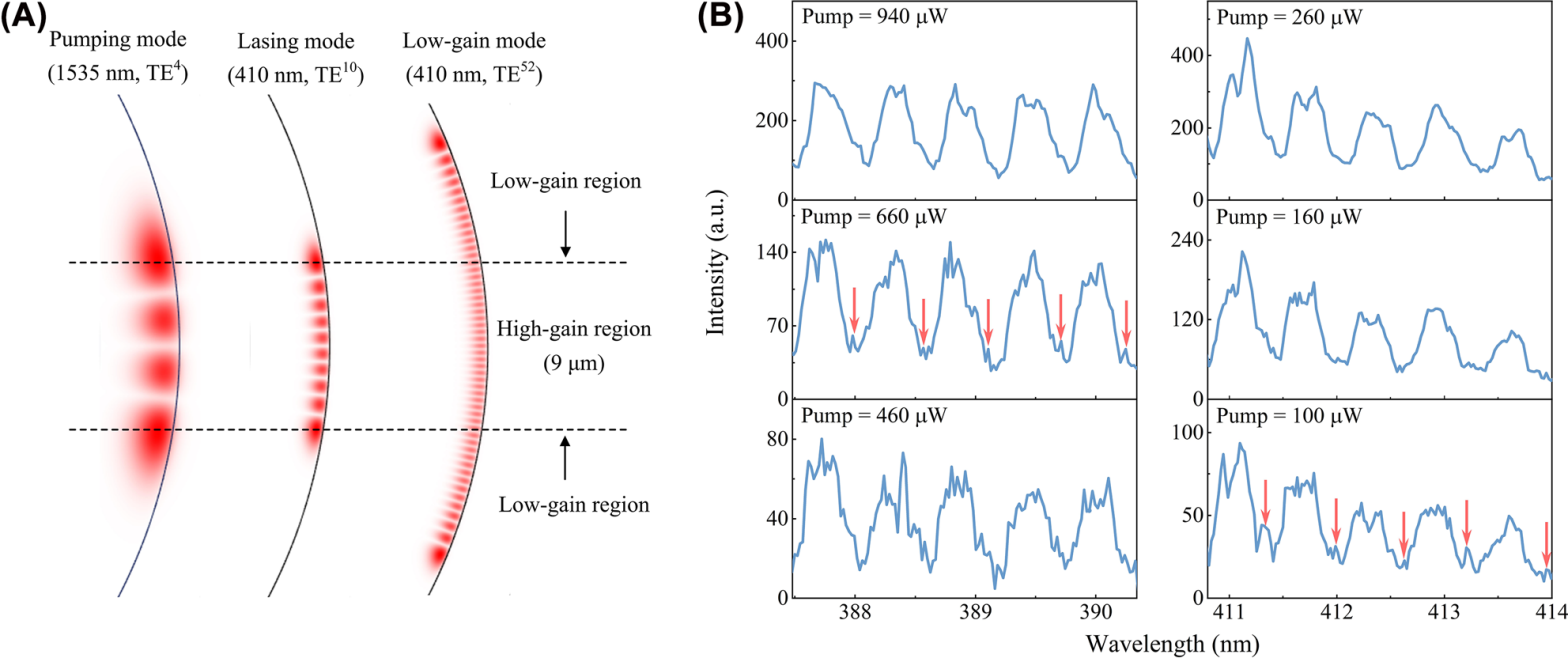
Annihilation of Purcell-enhanced spontaneous peaks. (A) Electric field intensity distributions of the pumping, lasing and low-gain modes on the cross-plane of the microsphere along the axis direction, where the superscript represents the axial quantum number and the dash lines are located at the maximum electric field intensities of the pumping mode. (B) Detailed lasing spectrum evolution with the increased pump power, where the red arrows with a spacing of one FSR point to typical Purcell-enhanced spontaneous peaks.

The effective gain from the gain materials is determined by the inversed populations and the spatial overlap between the emission modes and the inversed populations [30, 40]. And the distribution of the pumping mode determines the distribution of the excited states [22]. However, as the actual distribution of Er is difficult to be measured for this microsphere and the mode distributions in the near-infrared and ultraviolet bands are quite different, we cannot calculate an accurate spatial overlap between the emission modes and the inversed populations. Therefore, we roughly define the region between the two maximum electric field intensities of the pumping mode as the high-gain region. As shown in Figure 3A, the TE^10^ mode at 410 nm has a large spatial overlap with the high-gain region, achieving the desired lasing. In contrast, the TE^52^ mode at 410 nm distributes only 26.7% of its energy in the high gain region. Assuming that Er ions only exist in the H9/22 and I15/24 states and the Er ions in the high-gain region are completely inversed to the H9/22 states. The gain from the Er ions can be expressed by [22, 40–42]:

$$\begin{array}{ll} g_{\text{mat}} & =N\left(0.267\sigma_{em}-0.733\sigma_{ab}\right)\\ & =N\sigma_{ab}\left(0.267g_{1}/g_{2}-0.733\right)<0 \end{array}$$

where *N* represents the effective doping concentration, *σ*_*em*_ and *σ*_*ab*_ represent the emission and absorption cross-sections, respectively, *g*_1_ and *g*_2_ represent the level degeneracies of the I15/24 and H9/22 states. It can be found that *g*_mat_ is always negative, indicating that the TE^52^ mode at 410 nm cannot achieve lasing. However, the spontaneous emission will still be enhanced by the TE^52^ mode by using the Purcell effect [43, 44], leading to emerging of spontaneous emission peaks, corresponding to the resonance wavelength of the TE^52^ mode. Figure 3B is the detailed lasing spectrum evolution. At the low pump powers, there are many tiny peaks. These tiny peaks are recognized as the Purcell-enhanced spontaneous emission peaks, as they are arranged regularly with a spacing of one FSR. With the increased pump power, as the lasing modes increase with higher slopes, the tiny spontaneous emission peaks annihilate, remaining some major lasing peaks [45, 46]. This phenomenon should be also used to further confirm the lasing onset.

**Figure 4:**
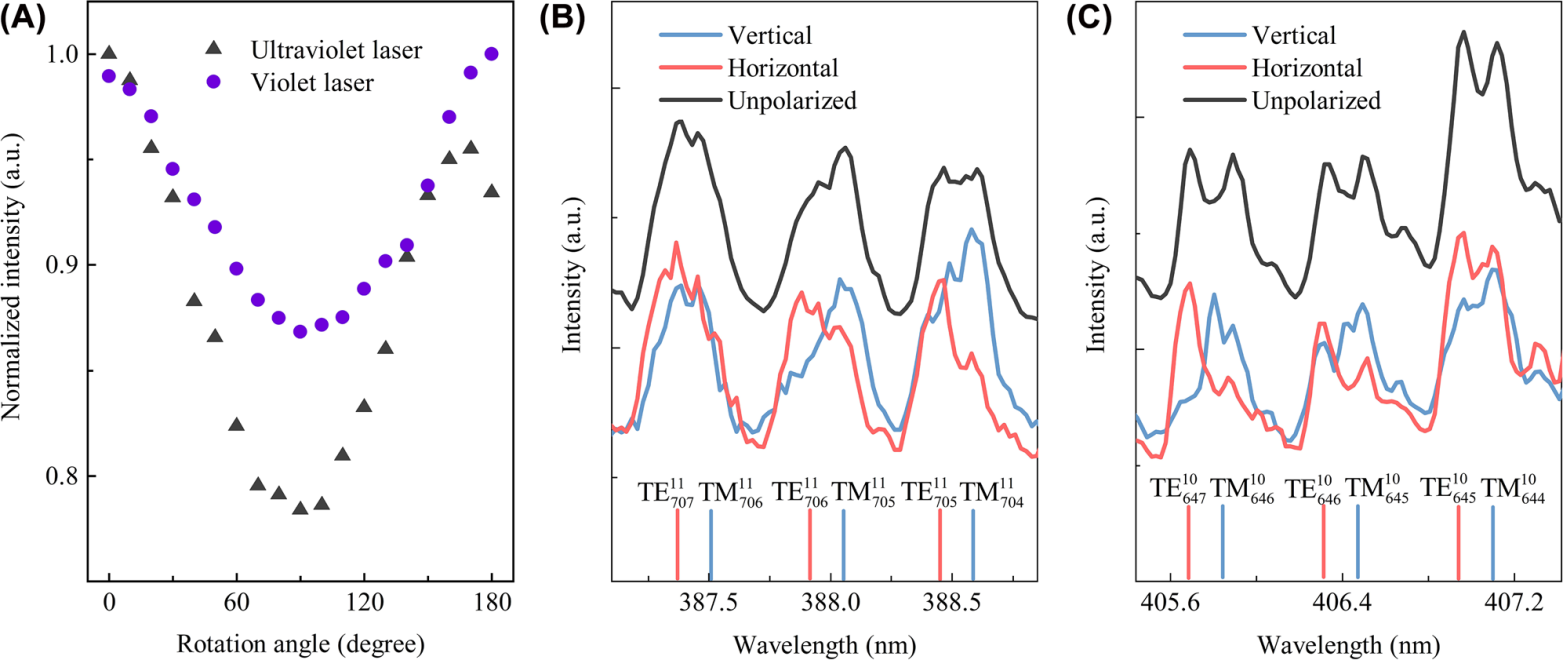
Laser polarizations. (A) Emission intensity as a function of the polarization degree. (B) and (C) Vertical, horizon polarized and unpolarized lasing spectra, with the calculated WGMs, where the superscript and the subscript represent the axial and azimuthal quantum numbers, respectively.

Polarization is another characteristic of a laser. Here, we place a linear polarizer before the spectrometer, and rotate it from 0 to 180° with a step of 10°. As shown in Figure 4A, the normalized intensity is approximately axial symmetry with respective to 90° and cannot reach zero, indicating they are elliptic polarized. Further plot of detailed polarized and unpolarized optical spectra at an excitation power of 2400 μW are shown in Figure 4B and C. The vertical and horizontal polarized lasers are easily distinguishable, as the lasing wavelength of the horizontal polarized laser is slightly shorter than that of the vertical polarized laser. It can be found that the unpolarized spectrum is composed of the vertical and horizontal polarized spectra, which is further matched by the theoretically calculated transverse magnetic (TM) and transverse electric (TE) polarized modes. The TE and TM modes with the same quantum number have similar mode distributions, therefore, their intrinsic loss from the surface scattering loss and optical gain from Er are also similar, which makes they lase simultaneously [47].

**Figure 5:**
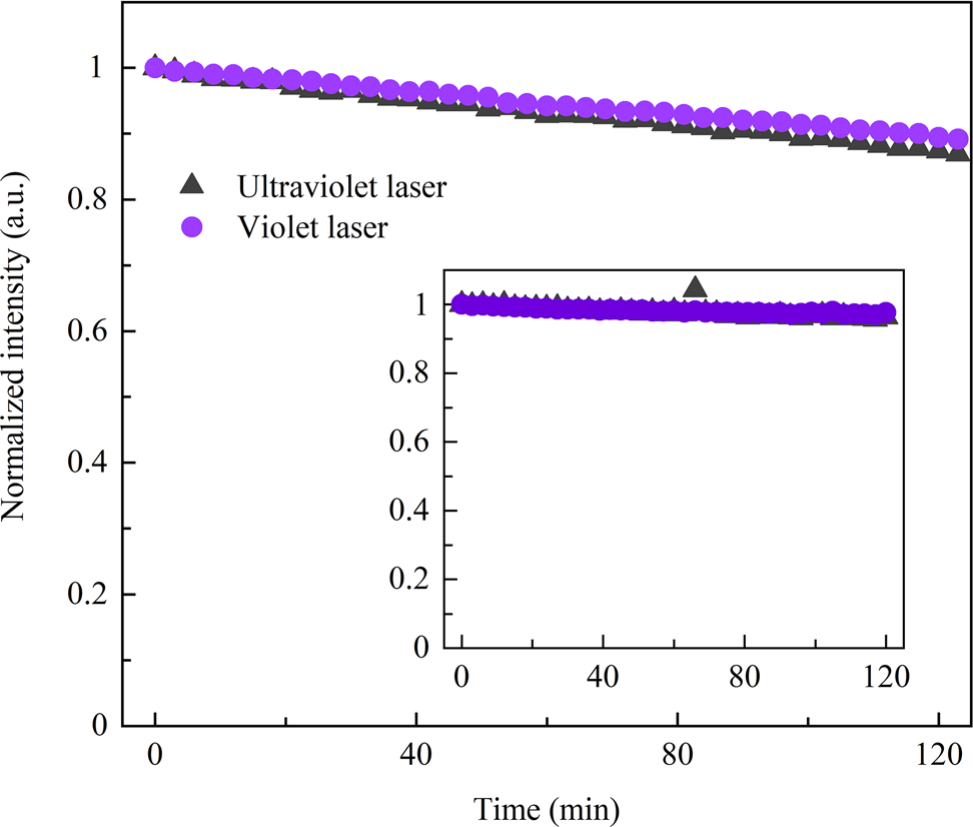
Intensity evolutions of the ultraviolet and violet lasers over the duration of 120 min. Inset: lasing intensity evolutions in another 70 μm-diameter microsphere fabricated with the same doping process.

The realization of short-wavelength multi-photon upconversion lasers usually suffer from the high pump density to inverse the populations and the extremely increased optical scattering loss at short wavelengths [48], which severely limits its stability for practical applications. From this point, a stable lasing intensity is essential for multi-photon upconversion process laser. As shown in Figure 5, the lasing intensities of the ultraviolet and violet lasers were measured over 120 min. Within the duration, the lasing intensities of the ultraviolet and violet lasers decline by 13 and 11%, which should be attributed to the damage of the gain materials or the dust induced contamination for the cavity. It is worth noting that, the inset shows the further lasing stability measurement for another microsphere, where both the ultraviolet and violet lasers did not degrade obviously (less than 5%).

## 3 Conclusion

In summary, we have demonstrated CW four- and five photon upconversion lasers by an Er-doped microcavity under room temperature, where the thresholds are as low as 176 and 600 μW, respectively. These are the highest order upconversion lasers based on rare-earth elements under CW laser pump at room temperature. The dependence of the lasing spectrum on the optical gain is further discussed. Low-gain resonance modes possessing low spatial overlap with the pumping mode introduce Purcell-enhanced spontaneous emission peaks, which annihilates after the lasing onset. Same-order TE and TM modes with the similar optical gain and loss achieve lasing simultaneously. Besides, this microlaser exhibits good stability of the lasing intensity over 120 min. This demonstration indicates a way to realize short-wavelength lasing by the high-order upconversion processes under CW near-infrared pump without requirement for rigorous phase match and ultrahigh pump power.

## Supplementary Material

Supplementary Material Details
