# Supplementary material for: Four- and five-photon upconversion lasing from rare earth elements under continuous-wave pump and room temperature
Source: Nanophotonics. 2022 Aug 15;11(18):4315–22. doi: 10.1515/nanoph-2022-0360 (PMC11501746; doi:10.1515/nanoph-2022-0360)
Supplement: Supplementary file 1 — Supplementary Material Details [file j_nanoph-2022-0360_suppl.docx]

# Supplementary Material for

# Four- and five-photon upconversion lasing from rare earth elements under continuous-wave pump and room temperature

Bo Jiang^1^, Yuchan Hu^1^, Linhao Ren^1^, Han Zhou^1^, Lei Shi^1,2,*^, and Xinliang Zhang^1,2^

^1^Wuhan National Laboratory for Optoelectronics, Huazhong University of Science and Technology, Wuhan 430074, China

^2^Optics Valley Laboratory, Wuhan 430074, China

*lshi@hust.edu.cn

**Keywords**: Whispering gallery mode, optical microcavity, rare earth, upconversion laser, multiphoton upconversion.

Ⅰ. Water induced absorption loss

We have measured the Q factor of a 56-μm-diameter undoped microsphere cavity under relative humidity of 55 %RH. As shown in Figure S1B, the linewidth of the resonance mode increases gradually. The increase of the linewidth can be caused by water in the atmosphere adhering to the cavity surface [1]. Since the water induced absorption loss is variable during the measurement and is lower than 0.4 MHz during 180 minutes, it is difficult to separate the water absorption loss from the total loss. Therefore, the water absorption loss is incorporated into the scattering loss. In Figure 2C, to obtain a more precise scattering loss, we have expanded the measured wavelength up to 1595 nm, in which the water absorption loss is relatively low.


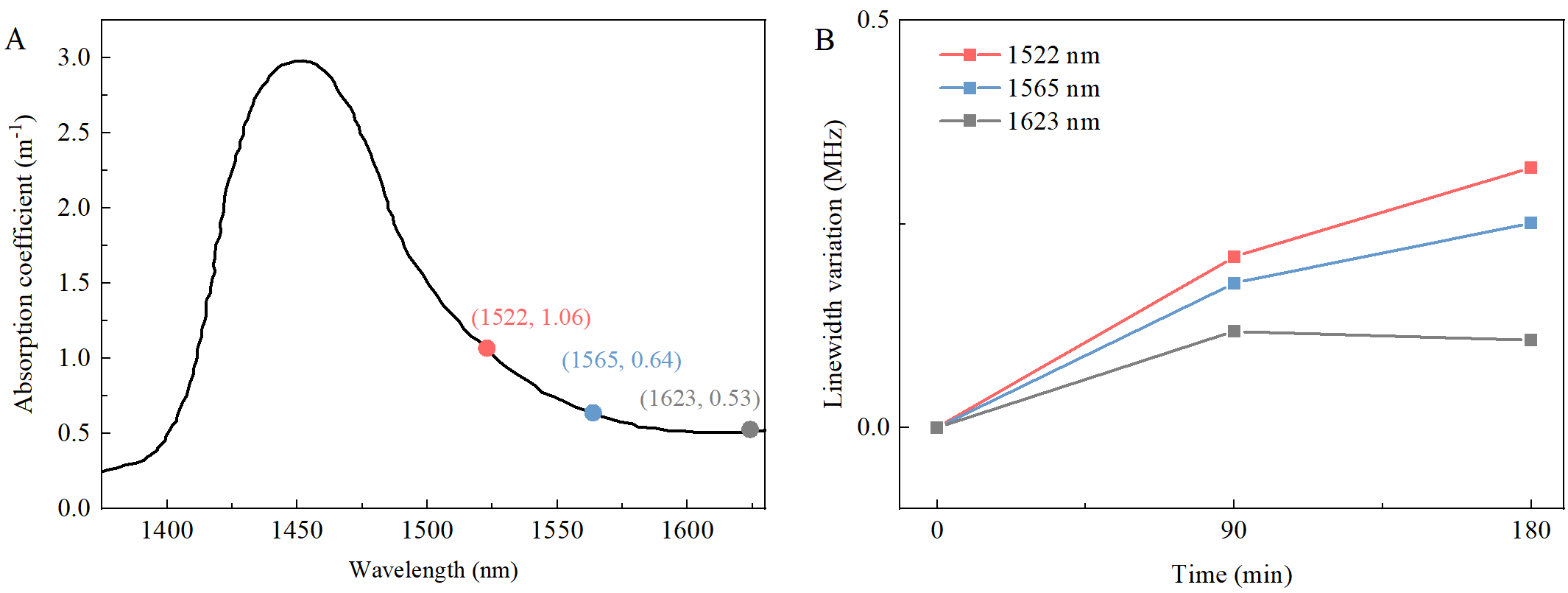


**Figure S1:** (A) Absorption coefficient of pure water versus the wavelength. (B) Linewidth variation during 180 minutes.

Ⅱ. Lasing threshold characterization in log-log scale

The lasing threshold in log-log scale corresponds to the maximum of the first-order derivative of the log-log curve [2]. As shown in Figure S2, in log-log scale, the lasing thresholds for the violet and ultraviolet bands are estimated to be 187 and 670 μW, respectively.


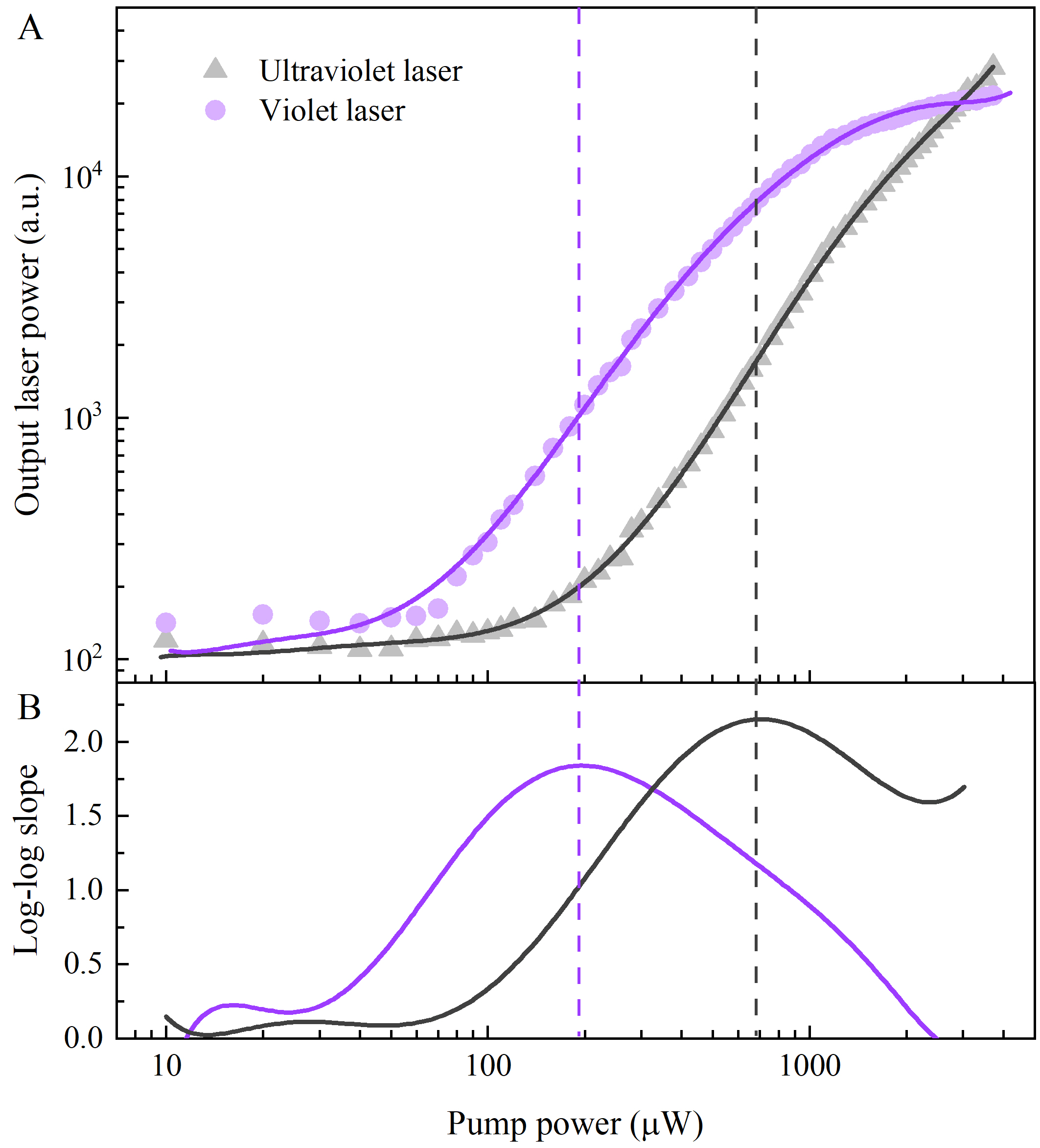


Figure S2: (A) Pump-output curves in log-log scale and polynomial fitting curves. (B) First-order derivatives of the polynomial fitting curves. The dashed lines correspond to the lasing thresholds.

Ⅲ. Er-based downshifting laser and nonlinear phenomenon

Figure S3 shows the optical spectra around the pump wavelength (1535 nm) band. It can be found that with an increased pump power, Er-based downshifting laser, Raman laser and four-wave mixing (FWM) are also observed. The thresholds for downshifting laser, Raman laser and FWM are estimated to be about 0.4, 430 and 560 μW, respectively.


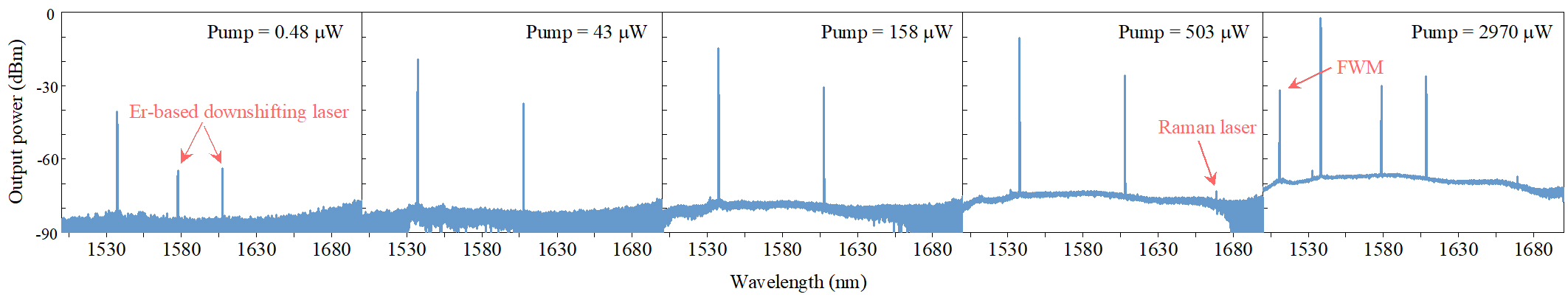


Figure S3: Optical spectra from 1500 to 1700 nm for the Er-doped microcavitysphere, with an increased pump power.

We fabricated a 64-μm-diameter undoped microsphere to compare their nonlinear effect. As shown in Figure S4, the Q factor of the undoped microsphere is estimated to be about 1.2×10^8^, which is close to that of the doped microsphere. As shown in Figure S5, the lasing threshold of Raman laser is estimated to be below 80 μW, which is far lower than that of the doped microsphere. Furthermore, the output power of Raman laser for the undoped microsphere reaches 4 μW under 163-μW pump power. In contrast, the output power of Raman laser for the Er-doped microsphere is only 200 pW under 2970-μW pump power. This phenomenon should be induced by the absorption of Er, which increases the threshold of Raman laser and lowers its output power. It is worth noting that, the FWM process requires quasi phase match, which should be very different for different microcavities. Therefore, Stokes Raman scattering with wide gain spectrum and no requirement for quasi phase match is used for this comparison.


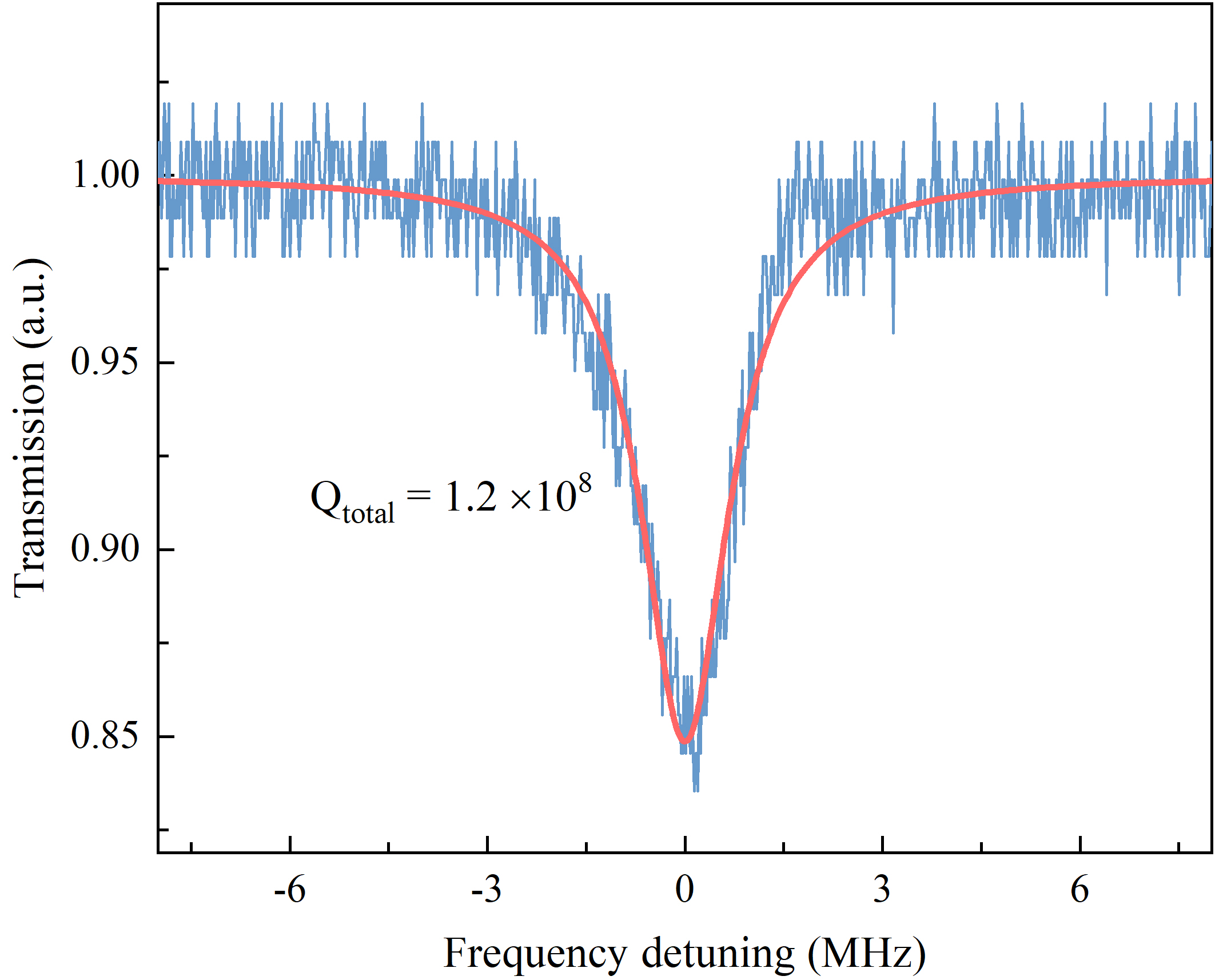


Figure S4: Transmission spectrum of the undoped microsphere operating at 1535 nm.


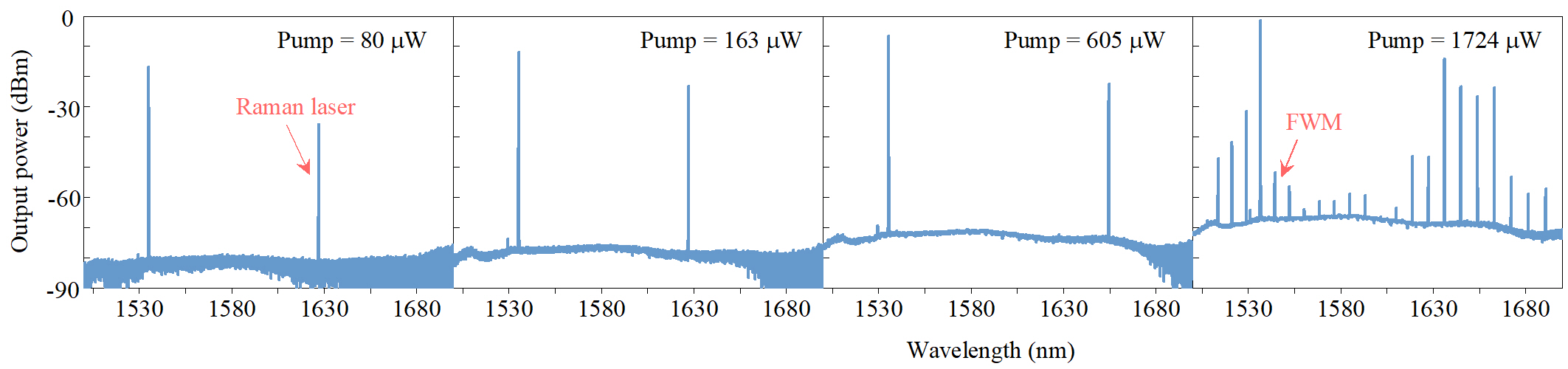


Figure S5: Optical spectra from 1500 to 1700 nm for the undoped microsphere, with an increased pump power.

**Supplementary References**

[1] S. Honari, S. Haque, and T. Lu, "Fabrication of ultra-high Q silica microdisk using chemo-mechanical polishing," *Appl. Phys. Lett.*, vol. 119, pp. 031107, 2021.

[2] C. Z. Ning, "What is laser threshold?" *IEEE J. Sel Top Quant.*, vol. 19, pp. 1503604, 2013.
